# On the potential for CO_2_ mineral storage in continental flood basalts – PHREEQC batch- and 1D diffusion–reaction simulations

**DOI:** 10.1186/1467-4866-13-5

**Published:** 2012-06-14

**Authors:** Thi Hai Van Pham, Per Aagaard, Helge Hellevang

**Affiliations:** 1 Department of Geosciences, University of Oslo, Pb. 1047, Blindern, Oslo, Norway

## Abstract

Continental flood basalts (CFB) are considered as potential CO_2_ storage sites because of their high reactivity and abundant divalent metal ions that can potentially trap carbon for geological timescales. Moreover, laterally extensive CFB are found in many place in the world within reasonable distances from major CO_2_ point emission sources.

Based on the mineral and glass composition of the Columbia River Basalt (CRB) we estimated the potential of CFB to store CO_2_ in secondary carbonates. We simulated the system using kinetic dependent dissolution of primary basalt-minerals (pyroxene, feldspar and glass) and the local equilibrium assumption for secondary phases (weathering products). The simulations were divided into closed-system batch simulations at a constant CO_2_ pressure of 100 bar with sensitivity studies of temperature and reactive surface area, an evaluation of the reactivity of H_2_O in scCO_2_, and finally 1D reactive diffusion simulations giving reactivity at CO_2_ pressures varying from 0 to 100 bar.

Although the uncertainty in reactive surface area and corresponding reaction rates are large, we have estimated the potential for CO_2_ mineral storage and identified factors that control the maximum extent of carbonation. The simulations showed that formation of carbonates from basalt at 40 C may be limited to the formation of siderite and possibly FeMg carbonates. Calcium was largely consumed by zeolite and oxide instead of forming carbonates. At higher temperatures (60 – 100 C), magnesite is suggested to form together with siderite and ankerite. The maximum potential of CO_2_ stored as solid carbonates, if CO_2_ is supplied to the reactions unlimited, is shown to depend on the availability of pore space as the hydration and carbonation reactions increase the solid volume and clog the pore space. For systems such as in the scCO_2_ phase with limited amount of water, the total carbonation potential is limited by the amount of water present for hydration of basalt.

## Introduction

Underground sequestration of carbon dioxide is a potentially viable greenhouse gas mitigation option as it reduces the release rate of CO_2_ to the atmosphere [1]. CO_2_ can be trapped subsurface by four storage mechanisms: (1) structural and stratigraphic trapping; (2) residual CO_2_ trapping; (3) solubility trapping; and (4) mineral trapping [2]. Mineral trapping has been considered as the safest mechanism in long-term storage of CO_2_[3].

Mineral storage of CO_2_ in basaltic rocks is favored over siliciclastic reservoirs both by the higher abundance of divalent metal ions in basalt and the faster reactivity of basaltic glass or crystalline basalt [4]. Moreover, basalts such as the Columbia River flood basalts (CRB) are abundant and in many places close to CO_2_ point source emissions [5]. During the last decade several flood basalts around the world have been mapped for the possibility of CO_2_ storage, and possible candidates such as CRB in USA and the Deccan traps in India have been identified [4-6].

To be a candidate for CO_2_ storage, the flood basalt must have a proper sealing and sufficient injectivity, the latter limited by the available connected pore space. In flood basalts, the connected pore space is typically found at zones containing abundant vesicles or in breccias between basalt flows. Because central zones of flood basalts commonly are dense and impermeable without vesicles, and flows are laterally continuous over large areas and commonly stacked vertically for hundreds of meters, flow units can act as seals [5]. The non-porous inner parts of flows may however be penetrated by networks of vertical fractures. These fractures can be open and conductive, or closed by mineralization and non-conductive.

**Figure 1:**
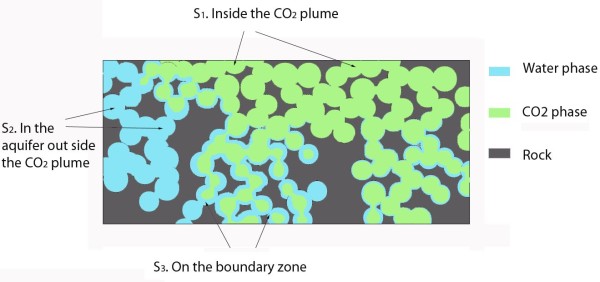
**Sketch of possible reaction settings during CO**_**2**_**storage in basalt.** System 1 (S1) is close to the injector and contains a wet CO_2_ (0.5 mole% H_2_O at 100 bar and 40 C) with no residual water; system 2 (S2) is fully in the H_2_O rich phase with CO_2_ diffusing in from the plume boundary; and system 3 (S3) is at the boundary of the CO_2_ plume with both sufficient non-wetting CO_2_ at a constant CO_2_ partial pressure of 100 bar and with sufficient water wetting the mineral surfaces and available for reactions.

The main objectives of this study were to performe batch- and 1D diffusion–reaction numerical simulations to determine the geochemical potential for secondary carbonate formation and to estimate the volume changes and the possibility of self-sealing following the basalt-CO_2_ interactions. The CRB system was used as an example case and our results were compared to earlier reported laboratory experiments and numerical simulations of CO_2_-basalt interactions. As CO_2_ stored underground will distribute spatially in the reservoir to give a range of reactive conditions, such as the potential of reactions by H_2_O dissolved in supercritical CO_2_[7,8] or reactions in the H_2_O-rich phase from residually trapped CO_2_, we divided the simulations into three systems representing different parts of CO_2_ storage: (1) basalt alteration in the H_2_O-rich phase at constant CO_2_ pressure; (2) basalt alteration in a H_2_O saturated CO_2_ phase, and (3) reactions at the boundary of the CO_2_ plume where CO_2_ diffuses into the aquifer from the boundary of the CO2 plume (Figure 1).

## Methods

All thermodynamic and kinetic calculations were performed using the geochemical code PHREEQC-2 [9]. This code is capable of simulating complex interactions between dissolved gases, aqueous solutions, and mineral assemblages in batch and 1D advection–diffusion-reaction mode. As the code can only model fully saturated systems, natural systems must be simplified to end-member situations, such as given by constant pressure boundary conditions as may be the case close to underground CO_2_ plumes, or the assumption of packages (batches) of water reacting along a reaction path with a homogenous sediment or rock body. Based on these limitations we divided the simulations into three systems representing different parts of CO_2_ storage: (1) basalt alteration in the H_2_O-rich phase at constant CO_2_ pressure; (2) basalt alteration in a H_2_O saturated scCO_2_ phase, and (3) reactions at the boundary of the CO_2_ plume where CO_2_ diffuses into the aquifer from the boundary of the scCO_2_ plume (Figure 1). In the second case, we assumed that the CO_2_ phase had swept through the systems and dried out residual water, giving only dissolved water in the scCO_2_ phase. In this case an upper limit of carbonation potential was estimated as reactions were allowed to occur until (nearly) all water was consumed, passing the upper 2 mol/Kgw theoretical limit for the Truesdell-Jones activity model [9].

The standard state adopted in this study for the thermodynamic calculations was that of unit activity for pure minerals and H_2_O at any temperature and pressure. For aqueous species other than H_2_O, the standard state was unit activity of the species in a hypothetical 1 molal solution referenced to infinite dilution at any temperature and pressure. For gases, the standard state was for unit fugacity of a hypothetical ideal gas at 1 bar of pressure. All simulations used the llnl.dat database based on the thermo.com.V8.R6.230 dataset prepared at the Lawrence Livermore National Laboratory, with additions of thermodynamic data for those phases not present (see description below).

CO_2_ fugacity coefficients were estimated according to the modified Redlich-Kwong (SRK) equation of state [10] and the solubility was adjusted for by a poynting correction term (*exp(v*_*CO2*_*(P*_*sat*_*- P)/RT)* where *v* denotes molar volume, *P* pressure, *R* the universal gas constant and *T* absolute temperature) [11]. The density of CO_2_ at 40 C and 100 bar was approximated from Bachu and Stewart [12] to be 600 Kg/m^3^ and the solubility of water in scCO_2_ at the same conditions was approximated to 0.5 mole% [13,14]

The simulations were divided into batch simulations of the H_2_O rich and CO_2_ rich phases respectively, and 1D diffusion of CO_2_ in the H_2_O rich phase to obtain information on the CO_2_-basalt interactions over a continuous range of CO_2_ pressures. The latter was solved by PHREEQC using $\partial_{t}C=D_{L}\partial_{x}^{2}C+q$, where *C* denotes molal (mol/Kgw) concentration, *q* denotes the sink term, subscripts *t* and *x* refer to derivatives in time and x-direction respectively, and an efficient diffusion coefficient *D*_*L*_ of 0.45x10^-9^ m^2^/s was used for CO_2_[15] and all solutes.

Dissolution rates of minerals in the basaltic rock were calculated according to a kinetic equation taking into account pH and the distance from equilibrium:

(1) $$\begin{array}{ll} r_{+}= & S\left\{k_{{}_{H}}\text{exp}\left(\frac{-E_{a,H}}{RT}\right)a_{H}^{{}^{n_{H}}}+k_{{}_{N}}\text{exp}\left(\frac{-E_{a,N}}{RT}\right)\right)\\ & \left(\;+k_{\mathrm{OH}}\text{exp}\left(\frac{-E_{a,OH}}{RT}\right)a_{H}^{{}^{n_{\mathrm{OH}}}}\right\}\left(1-\Omega \right) \end{array}$$

where *S* is the reactive surface area (m^2^), *k*_*i*_ are rate constants (moles/m^2^s), *a*_*H*_ is the H^+^ activity, *n* is the reaction order with respect to H^+^ and OH^-^, and Ω is the saturation state given by:

(2) $$\Omega =\exp\left(\frac{\Delta G_{r}}{RT}\right)$$

Where *ΔG*_*r*_ is the Gibbs free energy of the reaction, *R* is the gas constant, and *T* is absolute temperature. Reaction rate constants for crystalline basalt (pyroxenes and plagioclase) were obtained from Palandri and Kharaka [16], and pH dependencies were taken from the same source. The dissolution rate of basaltic glass was calculated according to the expression suggested by Gislason and Oelkers [17]:

(3) $$r_{+}=k_{+}\text{exp}\left(\frac{-E_{a}}{RT}\right)S{\left(\frac{a_{H^{+}}^{3}}{a_{Al^{3+}}}\right)}^{0.33}\left(1-\Omega \right)$$

where *k*_*+*_ is the far-from-equilibrium dissolution rate coefficient. The saturation state term 1-*Ω* was approximated to 1 (i.e., rate independent to distance from equilibrium) supported by earlier numerical estimates of glass-CO_2_ reactivity suggesting an approximately linear relation between time and reaction progress for basaltic glass [18]. This expression takes into account the effect of pH as well as the effect of the concentration of solutes such as fluoride as they complex with Al^3+^ and reduce the Al^3+^ activity [19]. The specific surface area for basalt (m^2^/g) was estimated by:

(4) $$S_{\mathrm{sp}}=\frac{\phi }{1-\phi }\frac{A_{p}}{{\bar{\rho }}_{s}V_{p}}$$

where the ratio A_p_/V_p_ denotes the ratio between pore surface and pore volume (m^-1^),  ϕ is connected porosity, and ${\bar{\rho }}_{s}$ (g/m^3^) is the density of the basalt solid estimated from the fraction of the individual basalt components. A *S*_*sp*_ value of 1.52 × 10^-5^ m^2^/g basalt (= 0.137 m^2^/Kg water) was obtained for the CRB using an average basalt solid density of 2.93 × 10^6^ g/m^3^ with 10% connected pore space and a *A*_*p*_*/V*_*p*_ ratio of 400 m^-1^[5]. The reactive surface area was calculated from the mass of the glass and minerals present according to:

(5) $$S_{i}=M_{i}n_{i}S_{\mathrm{sp}}X_{r}$$

where *M* and *n* are molar mass and moles of mineral *i*, and *X*_*r*_ is the fraction of the total mineral surface that is reactive. As *X*_*r*_ is highly uncertain and is suggested to vary by orders of magnitude [20,21], we used a value of 0.1 for the base case and varied *X*_*r*_ from 1 to 10^-3^. The use of mass or mass fractions of the individual basalt components to estimate the release rates of elements from the basalt is supported by a recent experimental study which suggests that release rates estimated from the sum of volume fractions of the constituent minerals are within one order of magnitude from measured values [22]. A list of kinetic parameters is given in Table 1. All secondary phases were allowed to form according to the local equilibrium assumption [23].

**Table 1:** **Kinetic parameters for dissolution of primary minerals based on empirical data given in Palandri and Kharaka**[16]**and for basaltic glass from**[17]

	**k**_**_H**_**(mol/m**^**2**^**s)**	**Ea**_**H**_** kJ/mol**	**n**_**H**_	**k**_**_N**_**(mol/m**^**2**^**s)**	**Ea**_**N**_**kJ/mol**	**k**_**_OH**_**(mol/m**^**2**^**s)**	**Ea**_**OH**_**kJ/mol**	**n**_**OH**_	**References**
Augite	1.58e-7	78	0.7	1.07e-12	78	-	-		[16]
Pigeonite	1.58e-7	78	0.7	1.07e-12	78	-	-		[16]
Feldspar	1.58e-9	53.5	0.541	3.39e-12	57.4	4.78e-15	59	-0.57	[16]
glass	1e-10	25.5	1	-	-	-	-	-	[17]
Magnetite	2.57e-9	18.6	0.279	1.66e-11	18.6	-	-	-	[16]

Changes in solid-phase volumes and porosities *ϕ* caused by the mineral reactions were calculated according to:

(6) $$\Delta \varphi_{t}=\left(1-\frac{\sum_{i}n_{i,t}{\bar{v}}_{i}}{V_{\mathrm{total}}}\right)-\varphi_{t=0}$$

where *ϕ*_*t=0*_ is the initial porosity, *n* and $\bar{v}$ are moles and molar volume of mineral *i* respectively, and *V*_*total*_ is the total volume of the system.

The basalt was defined to consist of a mixture of glass and crystalline basalt with mineral and glass fractions chosen based on reported data from CRB [6,24,25]. To represent the crystalline basalt, plagioclase (Ca_0.5_Na_0.5_Al_1.5_Si_2.5_O_8_) and the pyroxenes augite (Ca_0.7_Fe_0.6_ Mg_0.7_Si_2_O_6_) and pigeonite (Ca_1.14_Fe_0.64_ Mg_0.22_Si_2_O_6_) were chosen. The hydrolysis equilibrium constants of these phases were estimated using the PHREEQC program assuming ideal solid solutions of the end-members enstatite, ferrosilite and wollastonite for the pyroxenes, and albite and anorthite for the plagioclase. Equilibrium constants for the solid solutions for temperatures up to 100 C were estimated with PHREEQC and from these data coefficients *a* to *e* for the PHREEQC built-in analytical expression (*log K = a + bT + c/T + dlog*_*10*_*(T) + e/T*^*2*^) were estimated using non-linear regression in MATLAB.

The glass composition (Ca_0.015_Fe_0.095_ Mg_0.065_Na_0.025_ K_0.01_Al_0.105_ S_0.003_Si_0.5_O_1.35_) was taken from [6] and modified by adding a small fraction of sulfur which is a common minor constituent of the CR basaltic glass [26].

The secondary mineral assemblage was chosen based on reports on basalt weathering [27-30], with additional carbonates that could potentially form at elevated CO_2_ pressures from the release of Fe, Mg and Ca. The ankerite composition chosen for this work was CaFe_0.6_ Mg_0.4_(CO_3_)_2_ which corresponds to a solid solution of 0.6 ankerite (CaFe(CO_3_)_2_) and 0.4 dolomite (CaMg(CO_3_)_2_). Because ankerite (CaFe_0.6_ Mg_0.4_(CO_3_)_2_) was not listed in the thermodynamic database, we estimated values using the same approach as in [31]. The full list of secondary minerals is given in Table 2.

**Table 2:** Mineralogy included in the model

	**Initial Weight %**	**Density (g/cm**^**3**^**)**	^**2,3**^**Log K**^**0**^
**Primary minerals**			
^1^Augite (En0.35Fs0.3Wo0.35)	16	3.40	21.00
^1^Pigeonite (En0.57Fs0.32Wo0.11)	3	3.38	21.40
^1^Plagioclase (An50)	35	2.68	14.20
Glass Ca_0.015_Fe_0.095_Mg_0.065_Na_0.025_K_0.01_Al_0.105_ S_0.003_Si_0.5_O_1.35_	45	2.92	-99.00
Magnetite	1	5.15	10.47
**Secondary minerals**			
SiO_2_(am)	0	2.62	-2.71
Albite	0	2.62	2.76
Goethite	0	3.80	0.53
Calcite	0	2.71	1.85
Hematite	0	5.30	0.11
Kaolinite	0	2.60	6.81
Smec high Fe-Mg	0	2.70	17.42
Saponite-Mg	0	2.40	26.25
Celadonite	0	3.00	7.46
Stilbite	0	2.15	1.05
Dawnsonite	0	2.42	4.35
Siderite	0	3.96	-0.19
^1^Ankerite (Ank_0.6_Do_0.4_)	0	3.05	-19.51
Dolomite	0	2.84	4.06
Magnesite	0	3.00	2.29

The mineralogy of the CRB has been described in [25,32] and the weight fraction of pyroxene, feldspar and glass was estimated as average values from the reported data.

^1^Solid solutions. En (enstatite), Fs (ferrosilite), Wo (wollastonite), An (anorthite), Ank (ankerite), Do (dolomite). For details on the calculations of the ankerite solid solution see [31].

^2^Superscript ^0^ denotes Standard state (T = 298K, P = 1 atm). The equilibrium constant *log K* value is that for the forward dissolution reaction for one mole unit of the mineral.

^3^All thermodynamic data (*log K* and coefficients for the PHREEQC analytical temperature expression) from the llnl.dat PHREEQC database, except for the solid-solutions first estimated in PHREEQC by ideal solid solutions and then added to the PHREEQC database as new solid solution phases.

To simulate the CRB-CO_2_ interaction we used the average concentrations of solutes reported for the Grand Ronde Formation (Table 3). As supercritical CO_2_ (scCO_2_ at T > 31.1 C; P > 73.9 bar) is the preferred choice for CO_2_ storage, based on higher density compared to gaseous CO_2_, we simulated aqueous-phase basalt-CO_2_ interaction at a depth of 800 meters at a CO_2_ pressure of 100 bar and temperatures of 40 to 100 C. The reactivity of basalt and a H_2_O saturated scCO_2_ phase was simulated using an estimated 0.5 mol% H_2_O and a CO_2_ density of 600 g/cc giving an initial mass of 0.003 Kg H_2_O per 1 liter pore space.

**Table 3:** Composition of initial formation water

**Elements (totals)**	**Mol/kgw**
Na	1.0 x 10^-3^
Ca	6.0 x 10^-4^
K	1.0 x 10^-4^
Mg	2.0 x 10^-5^
Fe	1.2 x 10^-6^
Alkalinity (HCO_3_^-^)	2.0 x 10^-3^
Cl	3.0 x 10^-4^
S (SO_4_^2-^)	1.0 x 10^-4^
Si	2.0 x 10^-4^
Al	1.0 x 10^-6^
Log(O_2_)	-10.68
pH	7.5

## Results

### System 1: Basalt alteration in the H_2_O-rich phase at constant CO_2_ pressure

#### i) CRB mineral and glass dissolution and formation of secondary minerals

**Figure 2:**
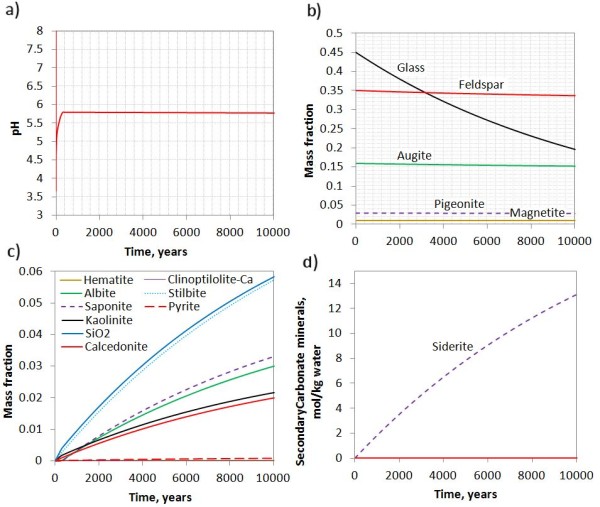
**Basalt alteration at 40 C and 100 bar CO**_**2**_**pressure over 10000 years. a**) pH changes; **b**) mass fractions of basaltic glass and crystalline basalt components; **c**) secondary phases formed; and **d**) moles of secondary carbonates (siderite) formed per kgw.

Following the injection of CO_2_ into the system, pH immediately decreased from 9.5 to below 4, and thereafter gradually increased to 5.8 at the end of 10000 years (Figure 2a). At the acidic pH secondary phases such as saponite (Ca_0.165_Mg_3_Al_0.33_Si_3.67_O_10_(OH)_2_), celadonite (KMgAlSi_4_O_10_(OH)_2_) and zeolite (stilbite) were thermodynamically stable and formed (Figure 2b). Glass dissolved orders of magnitude faster than the crystalline basaltic constituents and more than half dissolved after 10000 years (Figure 2c). The dissolution rate of glass was not increased by the aqueous fluoride as the Al^3+^ activity was fixed by the kaolinite and amorphous silica equilibria. The fluoride therefore only increased the total soluble aluminium. The steady release of Fe from the basalt saturated the water with respect to siderite and a total amount of 10 moles/kgw formed after 10000 years (Figure 2d). Other carbonates, such as ankerite, dolomite, magnesite, and dawsonite did not form as elements such as Mg and Ca was consumed by the non-carbonate secondary phases.

**Figure 3:**
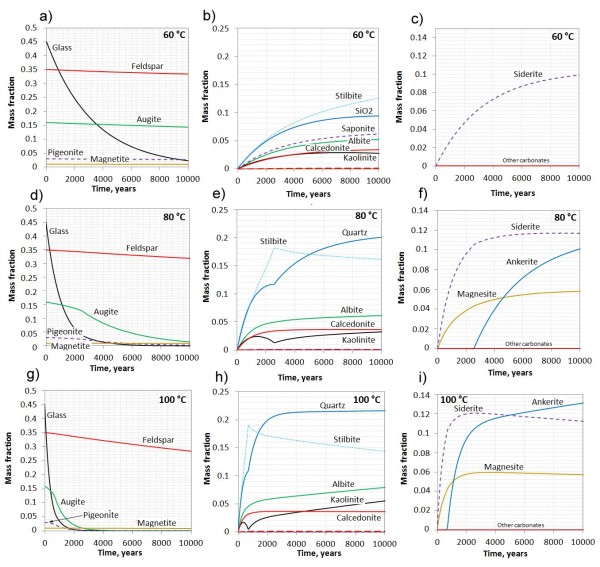
**Mass fractions of minerals following basalt alteration at 60, 80, and 100 C over 10000 years: a**, **d**, **g**) primary basalt minerals and glass; **b**,**e**, **h**) secondary phases except the carbonates; and **c**, **f**, **j**) carbonates.

**Figure 4:**
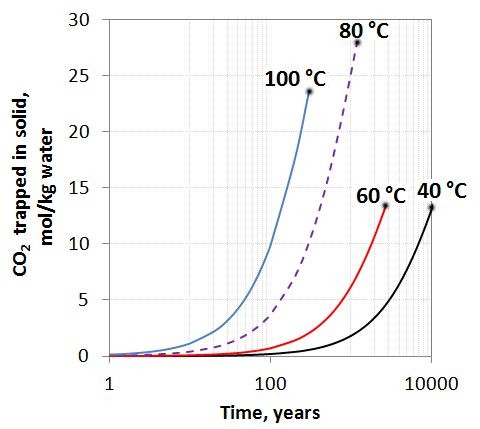
**CO**_**2**_**trapped in solids for 40 to 100 C simulations at 100 bar CO**_**2**_**pressure.** A cut-off value is used when all pore space is filled up with the secondary phases (see Figure 5). The simulations suggest that the total amount of secondary carbonates that form is dictated by the available pore space and the thermodynamic stability of secondary phases rather than temperature, whereas carbonate generation rates depend on the exponential increase of basalt dissolution rates with temperature.

The effect of temperature on the basalt hydration and carbonation was investigated by simulating the system at 60, 80 and 100 C (Figure 3). As for the 40 C simulation we see that basaltic glass dissolves orders of magnitude faster than the crystalline basalt components and the glass is the major source for the secondary phases. The dissolution rates of the basalt components scale exponentially with temperature, and the glass is almost completely dissolved after 10000 years at 60 C, whereas the time for a complete dissolution takes 4000 and 1500 years at 80 and 100 C respectively (Figure 3a, d, g). The secondary mineral assemblages were largely the same for all temperatures. Stilbite dominated together with amorphous silica (40 and 60 C) and quartz (80 and 100 C) (Figure 3b, e, h). Saponite formed at 40 and 60 C, but not at higher temperatures. Other secondary minerals such as albite, celadonite, and kaolinite formed at all conditions. At 60 C, magnesite and dolomite were still considered to be too slow to form (see [29]) and siderite was the only phase that formed. At 80 and 100 C, magnesite and later ankerite formed together with siderite. Taking zero porosity as the maximum extent of possible reactions we see that the total amount of CO_2_ trapped as solid carbonates did not change much with temperature (Figure 4). The reaction rates increased however with temperature and the time needed to reach the maximum potential therefore decreased with temperature (Figure 4).

#### ii) On the limitation of pore-space for the basalt carbonation

**Figure 5:**
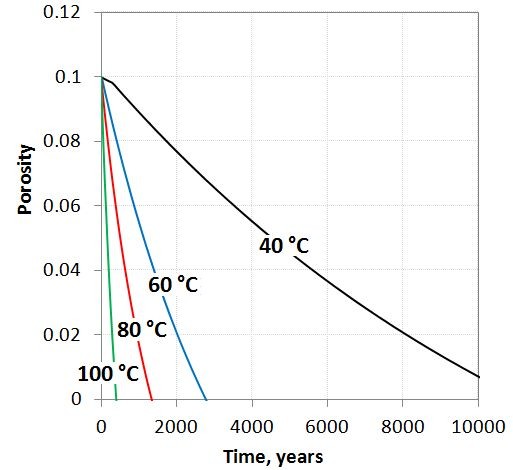
**Porosity changes caused by the basalt alteration at 40 to 100 C.** Secondary hydrated species and carbonates incorporate the H_2_O and CO_2_ masses into the solids and clogs the pore space. As reaction rates increase exponentially with temperature, the pore space is filled up faster at the higher temperatures.

Secondary phases such as stilbite and amorphous silica have lower density than the basalt components and alteration therefore leads to a reduction of pore space. At the presence of CO_2_, secondary carbonates further reduce the pore space. For the volume calculations we used expression (6) with the molar volumes listed in Table 2. At 40 C, the starting porosity of 10% is reduced to 0.85% after 10000 years. At the higher temperatures all porosity is lost after 2700, 1200, and 300 years respectively at 60, 80, and 100 C (Figure 5). Taking the extreme of 0% porosity as the limit for the reactions we obtain a maximum carbonation potential (mol CO_2_ stored/Kgw) at the different temperatures of 13.5, 29.3, and 28.5 moles for 60, 80, and 100 C (Figure 5). The simulated clogging of the pore space fits well with short-term laboratory percolation experiments on open-system basalt-CO_2_ alteration which shows loss of porosity and a rapid reduction of permeability during CO_2_-basalt interactions (e.g., [33]).

#### iii) Reduction of pore-space as a function of reactive surface area

**Figure 6:**
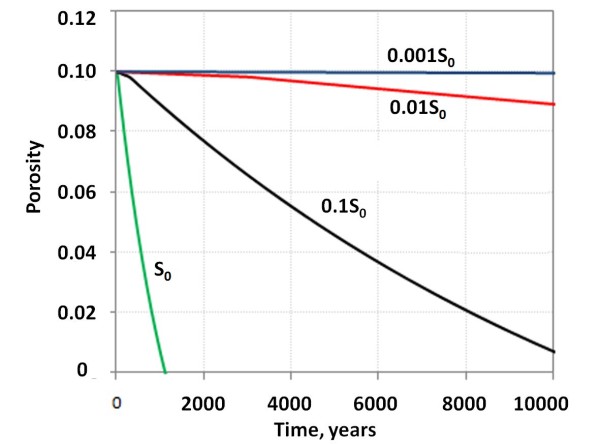
**Porosity changes caused by the basalt alteration at 40 C and specific surface areas ranging from S**_**o**_**(estimated total basalt surface area) down to a three orders of magnitude reduction.** The base case specific surface used was S_o_/10.

As the reactive surface area is a large uncertainty we simulated the changes of porosity over a range of values from a maximum being equal to the estimated physical surface area S_0_ (equation (5) with *X*_*r*_ *= 1*) to a three orders of magnitude reduction (Figure 6). The physical conditions of the simulated system was the same as for the base-case at 40 C and a CO_2_ pressure of 100 bars. At a reactive surface area that is equal to the estimated S_0_ all porosity is lost after approximately 1000 years as stilbite and siderite fills the pore space. If the reactive surface area is reduced by one order of magnitude (i.e. the base case) nearly 1/10 of the original 10% porosity is preserved. Further reductions by one and two orders of magnitude lead to smaller changes and at three orders of magnitude reduction relative to S_0_ almost no change is observed (Figure 6).

### System 2: The potential for carbonate growth in a H_2_O-saurated scCO_2_ phase

The reaction between H_2_O dissolved in scCO_2_ and basalt was simulated at 100 bar pressure and 40 C. The initial amount of water was 0.003 Kg and no H_2_O was allowed to enter the system. This is an ideal end-member case and serves to illustrate the carbonation potential in a volume with limited hydration potential.

**Figure 7:**
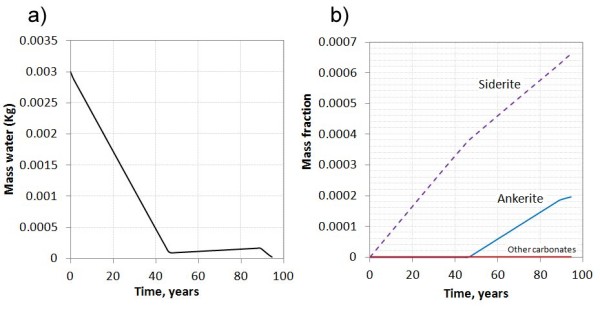
**Basalt alteration in the scCO**_**2**_**rich phase with initial 3 grams of water per liter pore space. A**) as zeolites form H_2_O is consumed and the water activity is reduced. After approximately 45 years most water is consumed, whereas all is gone before 100 years. **B**) siderite formed the secondary carbonate initially followed by ankerite.

As secondary phases such as stilbite formed, water was rapidly consumed and most gone after 45 years (Figure 7a). At this point stilbite was unstable and supplied water until all water was consumed after approximately 100 years (Figure 7a). Following the basalt hydration, siderite and ankerite formed from the released Ca, Mg, and Fe, with a final total amount of 0.2 moles CO_2_ consumed per liter pore space after 100 years (Figure 7b). If H_2_O had been allowed to dissolve into the scCO_2_ phase from residual aqueous phases trapped in the smaller pores, the carbonation potential would have been larger. This process is however, to the knowledge of the authors, not possible to simulate using the PHREEQC code, and was therefore outside the scope of this study.

### System 3: 1D diffusion of CO_2_ into the CRB aquifer

**Figure 8:**
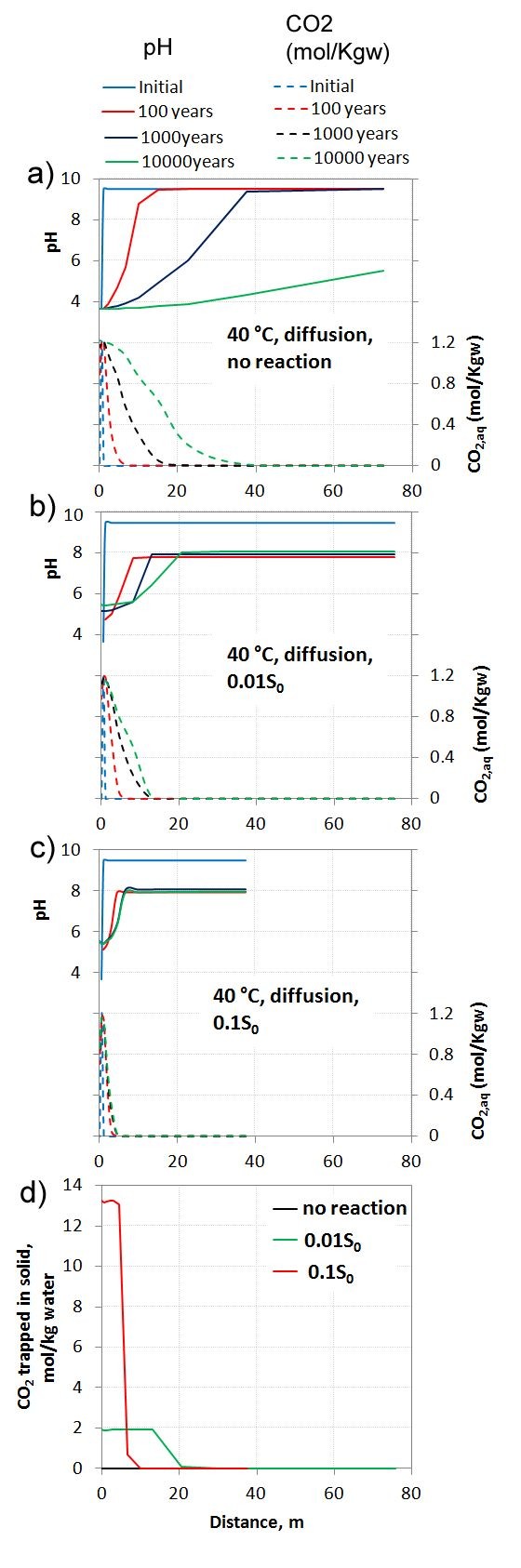
**1D reaction–diffusion of CO**_**2**_**into permeable basalt.** Partial pressure of the inlet boundary was fixed at 100 bar and with a column temperature of 40 C. As consumption of CO_2_ by siderite growth affects the depth of CO_2_ diffusion, we ran a sensitivity study on reactive surface area going from no reaction **(a)** up to the base case **(c)**. Finally the amount of CO_2_ trapped as solid carbonate (siderite) was compared for the base case and reduced reactive surface area **(d)**. We see that the reaction rates strongly constraint the depth of the column affected by the CO_2_ diffusion.

To see how the basalt reacted under different CO_2_ pressures, we defined a 1D diffusion–reaction simulation. This provided us with basalt-CO_2_ interactions over a continuous range of CO_2_ pressures from the background 1 bar up to the maximum 100 bars. The system corresponds to a stagnant zone presented as a column with one end close to the boundary of the injected CO_2_ plume and the other end further away from the plume (Figure 1). The distance reached for the CO_2_ into the column is given by the balance between diffusive transport and removal of carbon by secondary carbonate formation. We therefore varied reaction rates from no reaction giving the maximum lengtht of diffusive transport, and up to the base-case rate given by a reaction surface area 1 order of magnitude lower than the estimated physical surface area. Figure 8 shows pH, dissolved CO_2_ (mol/Kgw) and amount of secondary carbonate formed in the 1D column. As CO_2_ diffuses into the column pH drops to approximately 4 at full saturation. The depth of diffusion into the 1D column is approximately 40 meters at 1000 years if no carbonate forming reactions occur (Figure 8a). The penetration depth decreased rapidly if reactions were allowed as siderite formed and pulled carbon out of aqueous system (Figure 8b, c). At the highest reaction rate (base-case), CO_2_ diffused less than 10 meters into the column as approximately 13 moles/Kgw of siderite formed at the end of the 10000 years simulation (Figure 8d). Siderite formed at greater depth if the reactive surface area was reduced by another order of magnitude, but less formed in total (Figure 8d).

## Discussion

### Uncertainty on the reactive surface area

The reactive surface area is considered as a major source of uncertainty (e.g., [20,34]) and this leads to corresponding high uncertainties in timing and extent of reactions as dissolution rates have a first order dependence on reactive surface areas. Weathering rates in nature are commonly observed to be 1–3 orders of magnitude lower than in laboratory experiments (e.g., [20,21,34]), and this may in part be explained by differences in reactive and physical (total) surface area between experimental and natural systems. We assumed in this study a base-case reactive surface area 1 order of magnitude lower than the estimated physical surface area for the basalt. A further two orders of magnitude reduction in the reactive surface area, which is within the range of values expected for natural systems, resulted in little basalt alteration and only minor reduction of porosity (see Figure 5). A better understanding of the surface area of porous basalt and the effect of time (aging) on features such as dislocation densities and reactive surface areas are therefore required to understand the potential for CO_2_ mineral storage in basaltic rocks.

### Uncertainty on the choice of secondary phases used in the model

Growth rate experiments of carbonates such as magnesite and dolomite have shown that the activation energy is high and that growth is negligible at low temperatures (e.g., [35-37]). Dissolution rate studies of siderite suggests that the reaction rate is intermediate between calcite and magnesite [38,39], and growth rate data suggest that siderite may form down to room temperature [40]. Data on ankerite dissolution and growth is to the knowledge of the authors not known. The crystallographic and physical characteristics of ankerite do resemble those of dolomite and siderite, and the chemistry is related to dolomite with the Mg^2+^ substituted by various amounts of Fe^2+^ and Mn^2+^. If the growth rate is close to the magnesian carbonates such as dolomite and magnesite [41,42], the amount that may form during low-temperature alteration is likely low. In this case, more iron would be available for siderite growth. If on the other hand the growth rate is closer to siderite, we would expect ankerite or other FeMg solid solution carbonates to grow during low-temperature alteration.

One uncertainty related to the local-equilibrium assumption is on the growth retention time for the secondary carbonates. The local-equilibrium assumption predicts growth of the secondary phases as soon as an infinitesimally small supersaturation is reached [23]. The time it takes to nucleate sufficient mass to initiate a significant growth may however be hundreds to thousands of years for some secondary phases [31]. There are no nucleation rate data for siderite and ankerite and the retention time is hence unknown.

Finally, the total potential for secondary carbonate growth may be affected by the amount of magnesium and iron that enters ferromagnesian calcites. As a significant fraction of the metal cations may substitute for calcium (e.g., [43]), a iron-magnesium rich calcite may potentially form rather than ankerite and thereby reduce the amount of siderite formed.

### Comparisons to experiments, numerical simulations and natural analogues of basalt-CO_2_ interactions

Our simulations suggest that the potential for carbonate growth is limited to siderite or FeMg carbonates at low temperatures as secondary phases such as zeolites outcompeted the carbonates for calcium. We here compare our simulated results with reported data on CO_2_-basalt interactions from laboratory experiments, natural analogues, and other reported numerical simulations.

The reactivity of CRB and other continental flood basalts are available from the long-term (months to years) laboratory experiments done by Schaef and co-workers [6,24]. In these experiments basalt samples from USA, India, South Africa, and Canada were reacted with CO_2_ at about 100 bars and 60 to 100 C. Reacted samples from these experiments showed generation of Ca-rich carbonates interpreted as calcites with minor siderite and magnesite. In experiments on CRB using mixtures of H_2_S and CO_2_ at 60 C and 100 bar and run for 181 days, pyrite (FeS_2_) formed together with Mg-Fe poor calcite and a Ca-poor Fe-carbonate [6]. Our simulations at the same temperatures show rapid formation of siderite (60 C) or siderite and magnesite at higher temperatures (Figure 3). Our simulations do not predict any calcite growth as the calcium activity is lowered by zeolite formation. Calcite would however form in our models if the zeolites were not allowed to form at local equilibrium, and possibly if a magnesian ferroan (solid solution) calcite was used in the model instead of the pure end-member calcite. Therefore, the apparent difference between our model and the experiment may be caused by our use of the local equilibrium assumption, whereas the zeolites in the laboratory experiments did not form at low temperatures due to slow kinetics. Recent experiments on basalt dissolution support the preferential release of Mg and Fe over Ca at acidic conditions [22], suggesting that the MgFe-carbonates will dominate as secondary carbonates during CO_2_ storage in basalt.

Our numerical simulations share some similarities to other works such as by Marini [18] and Gysi [44], but our model and hence the outcome is different in several aspects. The most comprehensive work done earlier is the numerical simulations done by Marini [18] on the reactivity of crystalline and glassy CFB following CO_2_ storage. The initial mineralogy was similar to our study whereas the temperature of 60 C was slightly higher than our base case 40 C. In [18] the CO_2_-basalt interactions were stretched to last for more than 280000 years compared to our 10000 years perspective. The main differences between our model and [18] are on the choice of secondary mineral assemblage, and on the focus of limiting factors such as the availability of water for hydration in the present work. The lack of zeolites and hydrous phases other than kaolinite and goethite in [18] made Ca available for secondary carbonates and the total potential for carbonate formation was higher than in our work. Marini allowed dolomite and magnesite to form at 60 C, whereas our simulations only produced siderite at the similar conditions. Moreover, the formation of dawsonite in [18] is still uncertain and possibly limited at high silica activities and with an assemblage of stable NaAl-silicates defined to form [45]. Based on two different approaches, the reactive surface area for basalt was estimated to quite similar values. We estimated a specific surface area of approximately 1.5x10^-5^ m^2^/g_basalt_ (= 0.14 m^2^/Kg water at 10% porosity) based on the *A*_*p*_*/V*_*p*_ values estimated by [46] and reported in [5], and reduced this value by one order of magnitude to get the reactive surface area. Marini used a geometric model giving a reactive surface area of 0.41 m^2^/Kg water. The higher reactive surface area and higher temperature of [18] resulted in faster reactions and more rapid clogging of the pore space (within a few years). Studies of natural basalt systems at similar or higher temperatures may give some insight into how fast pore space is clogged by basalt hydration or carbonation, and this should be used to improve the estimates of reactive surface areas of basalt for future studies.

Another numerical study on low-temperature (25 C, 30 bar CO_2_) basaltic glass alteration was presented by Gysi et al. [44]. Again a main difference is on the choice of secondary minerals. Gysi et al. [44] allowed dolomite, magnesite, and Fe-Mg carbonate to form together with calcite and siderite, whereas we did not allow other Mg-Fe carbonates to form than ankerite. As previously stated, the low-temperature formation of dolomite and magnesite is not likely because of the high apparent activation energy and small kinetic coefficients for the growth of Mg-carbonates [35-37]. Other carbonates such as siderite and potentially FeMg-calcites are more likely to form at these low temperatures. The high reactive surface area used in [44] is based on a geometric model for glass fragments, and is hence not directly comparable with the surface area estimated for a vesicle pore space of a solid basalt. Although no inverse modeling was done to estimate the reactive surface area of the basalt in [44], fragmented basaltic rocks such as hyaloclastite breccias are expected to have significantly higher reactive surface areas than porous solid basalts, and they are therefore correspondingly more reactive.

One example of a natural analogue that shed light on CO_2_ basalt interactions is the CO_2_ charged basalt hosted groundwaters at Hekla, Iceland. Solution aqueous species sampled from natural cold springs and rivers here showed a drop in total inorganic carbon (TIC) that was interpreted to result from considerable formation of secondary carbonate phases such as calcite [47]. Reaction path modeling of the system suggests however that the carbonate formation is associated with high pH in accordance with the low TIC in the sampled waters. This system is therefore different from basalt CO_2_ storage projects where higher CO_2_ pressures may be maintained over time and the pH is lower. In addition to calcite, dolomite was also suggested as a potential storage host for the low temperature reactions in Hekla [47]. This may however be questionable as long-term laboratory experiments at room temperature have failed to form dolomite even at significant super saturations [48], explained by the high activation energy for dolomite growth [32,41]. Another natural analogue that more closely corresponds to industrial CO_2_ storage is the basalt-hosted petroleum reservoir on Nuussuaq, West Greenland. In this system the bulk carbonate formation appears to have occurred as secondary weathering products. Other alteration products such as zeolites and oxides were replaced by dolomite, magnesite, siderite, and calcite at temperatures of 70–120 C [49]. Therefore, taking into account the basalt weathering products and not only primary basalt minerals appears to be vital in estimating the total potential for secondary carbonate formation and the long-term potential for CO_2_ storage in basalt systems.

## Summary and conclusions

Simulations of closed-system (P_CO2_ = 100 bar, 40 C) and 1D reaction–diffusion (P_CO2_ = 0–100 bar, 40 C) alteration of basalt suggest that the potential of secondary carbonate formation is limited to siderite at low temperatures as divalent metal cations are preferentially consumed by zeolites and oxides. Higher temperatures 60 – 100 C appear to be in favor of secondary carbonate formation, allowing the precipitation of carbonates such as magnesite, siderite and possibly dolomite and other FeMg carbonates (ankerite). Given an unlimited source of CO_2_ (fixed CO_2_ pressure), the total amount of CO_2_ stored as solid carbonates is orders of magnitude higher than the 1–2 mol/Kg water solubility in the formation water (Figure 4). The total amount trapped might however be reduced if CO_2_, H_2_O or pore space are limiting factors. The formation of secondary hydrous and carbonate phases increases the volume of solids and the porosity is correspondingly reduced (Figure 5). This together with the immobilization of CO_2_ by solid carbonate formation is in favor of safe long-term storage of CO_2_ in basaltic aquifers.

## Competing interests

We have no competing interests with any organization to publish the manuscript ‘On the potential for CO2 mineral storage in continental flood basalts – PHREEQC batch- and 1D diffusion-reaction simulations‘.

## Authors’ contributions

VTH Pham has made substantial contributions to conception and designs the manuscript. Her contributions are to acquisition of data, analysis and interpretation of data. PA did revising and has given final approval of the version to be published. HH involved in drafting the manuscript, writing methodology part and revising of the final version. All authors read and approved the final manuscript.
